# Physicochemical Characterization of *In Vitro* LDL Glycation and Its Inhibition by Ellagic Acid (EA): An *In Vivo* Approach to Inhibit Diabetes in Experimental Animals

**DOI:** 10.1155/2022/5583298

**Published:** 2022-01-19

**Authors:** Saheem Ahmad, Sultan Alouffi, Saif Khan, Mahvish Khan, Rihab Akasha, Jalaluddin Mohammad Ashraf, Mohd Farhan, Uzma Shahab, Mohd Yasir Khan

**Affiliations:** ^1^Department of Clinical Laboratory Sciences, College of Applied Medical Sciences, University of Hail, Saudi Arabia; ^2^Molecular Diagnostic & Personalized Therapeutic Unit, University of Hail, Saudi Arabia; ^3^Department of Basic Dental and Medical Sciences, College of Dentistry, University of Hail, Ha'il 2440, Saudi Arabia; ^4^Department of Biology, College of Science, University of Hail, Ha'il 2440, Saudi Arabia; ^5^Department of Clinical Biochemistry, College Applied Medical Sciences, Jazan University, Jazan 45142, Saudi Arabia; ^6^Department of Basic Sciences, King Faisal University, P.O. Box 400, Al-Ahsa 31982, Saudi Arabia; ^7^Department of Biotechnology, Khwaja Moinuddin Chishti Language University, Sitapur-Hardoi Bypass Road, Lucknow 226013, India; ^8^Department of Biotechnology, School of Applied & Life Science (SALS), Uttaranchal University, Dehradun, 248007 Uttarakhand, India

## Abstract

Hundreds of millions of people around the globe are afflicted by diabetes mellitus. The alteration in glucose fixation process might result into hyperglycaemia and could affect the circulating plasma proteins to undergo nonenzymatic glycation reaction. If it is unchecked, it may lead to diabetes with increase in advanced glycation end products (AGEs). Therefore, the present study was designed to inhibit the diabetes and glycation by using natural antioxidant “ellagic acid” (EA). In this study, we explored the antidiabetes and antiglycation potential of EA in both *in vitro* (EA at micromolar concentration) and *in vivo* systems. The EA concentrations of 10 and 20 mg kg^−1^B.W./day were administered orally for 25 days to alloxan-induced diabetic rats, a week after confirmation of stable diabetes in animals. Intriguingly, EA supplementation in diabetic rats reversed the increase in fasting blood sugar (FBS) and hemoglobin A1c (HbA1c) level. EA also showed an inhibitory role against glycation intermediates including dicarbonyls, as well as AGEs, investigated in a glycation mixture with *in vitro* and *in vivo* animal plasma samples. Additionally, EA treatment resulted in inhibition of lipid peroxidation-mediated malondialdehyde (MDA) and conjugated dienes (CD). Furthermore, EA exhibited an antioxidant property, increased the level of plasma glutathione (GSH), and also helped to decrease histological changes evaluated by histoimmunostaining of animal kidney tissues. The results from our investigation clearly indicates the antiglycative property of EA, suggesting EA as an adequate inhibitor of glycation and diabetes, which can be investigated further in preclinical settings for the treatment and management of diabetes-associated complications.

## 1. Introduction

The worldwide prevalence of type 2 diabetes mellitus (T2DM) and its associated complications resulting from insufficiency of insulin secretion and resistance have increased noticeably and tremendously contribute to the global burden of disability and mortality [1]. Hyperglycaemia causes damage to those cell types that are able to maintain sugar level, strongly determining the rate of nonenzymatic glycation. The glycation process proceeds through several stages and ameliorates oxidative as well as reactive carbonyl species (RCS) stress which accommodate advanced glycation end product (AGE) accumulation intriguingly responsible for long-term acute ailments of diabetes [2]. The covalent bonding between the carbonyl group of reducing saccharide and the side chains of amino groups of proteins results in Schiff's bases and more stable Amadori products, which are named as early glycation products [3]. Furthermore, subsequent irreversible reactions leads to the formation of highly reactive glycation intermediates known as reactive carbonyl compounds mainly glyoxal (GO), methylglyoxal (MG), and 3-deoxyglucosone (3-DG). These reactive carbonyl compounds can induce more stable heterogeneous advanced glycation end products called AGEs. Therefore, diabetes accelerates AGE formation and its gradual build-up in circulation and body tissues [4]. This phenomenon is strongly associated with renal afflictions [5], osteoarthritis [6], and atherosclerosis [7] with diabetes.

LDL glycation was first described by Schleicher et al. [8]. The LDL particles have protein(s) on their surface that determine their interactions with cell-membrane receptors. The core of LDL particles contains esterified cholesterol, triglyceride, and other lipids. Under abnormal LDL accumulation in plasma, the extent of LDL glycation varied as a function, both of the duration of LDL incubation and of the concentration of reactive carbonyl in the incubation mixture [9]. The increased glycative stress induces the impaired uptake of glycated LDL by the LDL receptor of the liver and the scavenger cells, increasing plasma half-life of the LDL molecule. Glycation causes damage to LDL and other plasma proteins that might serve as a predictor for early detection of diabetes. Our previous study revealed that MG-modified glycated LDL might be a potential target for circulating autoantibodies against glycated LDL, which increases with the increase in duration of type 2 diabetes mellitus [10]. The autoantibodies that react with glycated LDL generates both intra- and extravascular immune complexes, which may further contribute to other immune factors involved in the destruction of *β*-cells of the pancreas [11]. People with diabetes undergo *in vivo* glycation of LDL apolipoprotein B (Apo B) which gave rise to the hypothesis that lipoprotein glycation contributes to the diabetes and its associated complications [9, 12].

At present, many patented drugs are well known to circumvent the growing effect of AGEs, including aminoguanidine (AG), pyridoxamine, and other antidiabetic drugs [5, 13, 14]. The action mechanisms of these drugs impede the formation of RCS and AGEs, lowering of cholesterol and triglyceride levels, when applied at higher concentrations [15–17]. In the case of AG treatment, the higher concentration is not preferred as it reacts with vitamin B6 causing its deficiency and inducing toxicity [18]. Therefore, the need of the hour is to screen out natural antiglycating agents that are nontoxic with longer half-life to potentiate inhibition of early (Amadori), intermediates (carbonyl content-dicarbonyls), and AGEs [19]. The number of aqueous plant extracts of dietary spices, fruits, vegetables, and herbs has been evaluated and used as an inhibitor of AGE formation. Polyphenols are reported to have some health benefits, which are linked to their antioxidant properties, and AGE inhibition activity suggests that they exert their inhibitory activity by interrupting the autoxidative pathways [20].

Ellagic acid (EA), which is a potent antioxidant and dimeric derivative of gallic acid (GA), includes hydrophilic moiety composed of four hydroxyl and two lactone groups and a lipophilic planar fragment, consisting of two hydrocarbon phenyl rings. The EA can be found as a polyphenolic compound present in many berries and pomegranate at relatively high concentrations [20]. In this study, EA was used as an inhibitor of *in vitro* glycation parallel with *in vivo* glycation inhibition in a diabetic animal model to evaluate its potential as an antiglycating and antidiabetic compound which might turn out as a silver lining. Furthermore, *in vivo* study depicts an activity of EA which mediates a preventive role against AGE accumulation-mediated histological changes due to immunoreactivity of AGEs-IgG in the globular basement membrane (GBM) of the kidney in a diabetic rat model. Therefore, the aim of this study is to repurpose the EA compound to elucidate its antiglycative as well as antidiabetic property by inhibiting the loss of the biomolecular function.

## 2. Materials and Methods

Low-density lipoprotein (Human-LDL), paraformaldehyde, thiobarbituric acid, ellagic acid (EA), nitro-blue tetrazolium (NBT), dimethyl sulfoxide (DMSO), methylglyoxal (MG), aminoguanidine (AG), Tris-citrate buffer, phosphate buffer saline (PBS), alloxan, guanidine hydrochloride, malondialdehyde, and anti-CML ELISA kit were obtained from Bioassay Technology Laboratory. Fluorescein isothiocyanate conjugate- (FITC-) labelled IgG was obtained from Calbiochem (USA).

### 2.1. Physicochemical Characterization of Glycation

Human low-density lipoprotein (LDL) was isolated from the blood plasma from a normolipidemic donor [21]. *In vitro* LDL glycation and its inhibition were characterized by UV-Vis spectroscopy at 282 nm wavelength described previously [22, 23]. The effect of ellagic acid (EA) as an antiglycating agent at varying concentrations (80, 130, and 180 *μ*M) in comparison with 10 mM aminoguanidine (AG) were examined *in vitro*. EA was dissolved in 10% DMSO with homogenate saline. EA is almost insoluble in acidic media and distilled water, while its water solubility is significantly improved by basic pH.

The rate of ketoamine formation is directly proportional to the fructosamine concentration and is measured by the spectrophotometric technique. In the absence and presence of EA in the glycation mixture, the degree of LDL glycation and glycation inhibitory action of EA was observed by detecting ketoamine moieties (NBT assay) at 525 nm wavelength and carbonyl content detection by using 2,4-dinitrophenylhydrazine (DNPH) at 360 nm wavelengths [24]. The content of ketoamine moieties and carbonyl content (nM mL^−1^) was estimated by using an extinction coefficient which is 12640 M^−1^ cm^−1^ and 22,000 M^−1^cm^−1^. However, effect of EA propensity for AGEs and intrinsic fluorescence of glycated LDL was subjected to fluorescence spectroscopy as described previously [22, 25, 26]. The excitation wavelength and emission wavelength range for AGE detection were 370 nm and 400-600 nm, respectively. To detect EA effect upon aromatic amino acid environment that gives intrinsic fluorescence, the samples were excited at 295 nm and the emission was observed in the range between 310 and 450 nm wavelength [27].

### 2.2. Agarose Gel Electrophoresis

The agarose gel electrophoresis of LDL was done according to Noble with slight modification [28]. The barbital buffer contained barbituric acid, 0.05 M, with pH 8.6 maintained with sodium hydroxide. The barbital buffer was used as a running buffer and 0.8% agarose was dissolved in 50 mL as described previously.

### 2.3. Ethical Statement for Animals and *In Vivo* Study

To conduct an animal study *in vivo*, Wistar rats (male) weighing around 200-250 g were acquired from CDRI, Lucknow, India. After acclimatization of animals, per cage, five rats were housed in a temperature-controlled room with 12 h light/dark cycles with accessibility to food and water ad libitum and experimented with care to minimize the pain. The implicated protocol of intervention for animal study approval was given by the Institutional Animal Ethical Committee (IAEC) of Integral University, Lucknow, India, with approval No: IU/IAEC/16/05.

### 2.4. Development of Diabetic Animal Model (*Wistar Rats*)

Alloxan which is chemically known as 5,5-dihydroxyl pyrimidine-2,4,6-trione is an organic compound and glucose analog which has the molecular formula C_4_H_2_N_2_O_4_ and a relative molecular mass of 142.06. Alloxan is one of the common diabetogenic agents often used to assess the antidiabetic potential of both pure compounds and plant extracts in studies involving diabetes [29]. After being acclimatized for 1 week, animals were randomized and divided into four groups, and each group contained five rats (*n* = 5). In group I, normal control rats received 0.9% saline while groups II–IV were fasted for 12 hours prior to intraperitoneal administration of alloxan (65 mg kg^−1^dissolved in 0.9% saline) to develop diabetes in rats. The fasting blood sugar (FBS) of alloxan-administered group II-IV rats was observed for four weeks (one month) by using a one-touch glucometer. After one month of alloxan-induced diabetes, rats with an increased FBS level (>150 mg dL^−1^) were selected for treatment with EA (groups III–IV) [30]. The selected rats in group II were diabetic rats with no EA treatment while group III and IV diabetic rats were treated with EA (10 and 20 mg kg^−1^, respectively). The animals in the control group received the basal diet only.

### 2.5. Toxicity Study and Dose Preparation of EA

To check toxicity of EA, single oral dose administration of EA at different concentrations (50, 100, and 150 mg kg^−1^ body weight (b.w.), intragastrically) was given simultaneously to the animals [31]. EA dissolved in 10% of dimethyl sulfoxide (DMSO) homogenized with saline. Intragastric intubation (0.3 mL) of EA with an oral dose of 10 and 20 mg kg^−1^ b.w./day for a period of 25 days was selected on the basis of previously published reports [32, 33].

### 2.6. Estimation of FBS and HbA1C from EA-Treated Diabetic Rat *In Vivo*

The blood was taken through cardiac puncture and collected in heparin-coated tubes. FBS and HbA1c values were measured in the whole blood [34]. FBS was monitored with a one-touch glucometer. HbA1c was determined by using a commercial kit from Bio-Rad to perform HbA1c test using the HPLC-based D-10 HbA1c program. Plasma separation from groups I–IV was pooled, aliquoted, and stored either at 4°C or −20°C for further biochemical parameters to study.

### 2.7. Biochemical Assessment in Animal Plasma

The plasma of control, one-month-old diabetic, and diabetic EA-treated animals was assayed to trace early, intermediate, and advanced glycation products. Plasma ketoamine moieties (NBT-assay) and carbonyl content were estimated by taking the absorbance at 525 nm and 360 nm wavelengths through UV-Vis spectroscopy. However, the fluorescence spectroscopy to detect AGEs was done by the methods described above [35]. Plasma-reduced glutathione (GSH) level estimated by using 5,5′-dithio-bis-[2-nitrobenzoic acid] (DTNB) was done as per the protocol described by Sedlak and Lindsay with slight variation [36]. Investigation of conjugated diene (CD) and malondialdehyde (MDA) from lipid fraction of plasma was done as discussed in previous studies [22, 37].

### 2.8. CML Detection by ELISA

CML is a nonfluorescent AGE precursor. CML detection was done in sera by using an anti-CML ELISA kit (Bioassay Technology Laboratory). The ELISA kit provides rapid detection and quantitation of CML protein adducts. The composed in vitro glycation and EA-antiglycation mixture was subjected to anti-CML ELISA. In vivo CML detection from the control, diabetic, and diabetic EA-treated serum samples was also subjected to ELISA for detection of CML. The absorbance was measured at 450 nm.

### 2.9. Immunohistochemistry of Diabetic and EA-Treated Rat Kidney Tissues

**Scheme 1:**
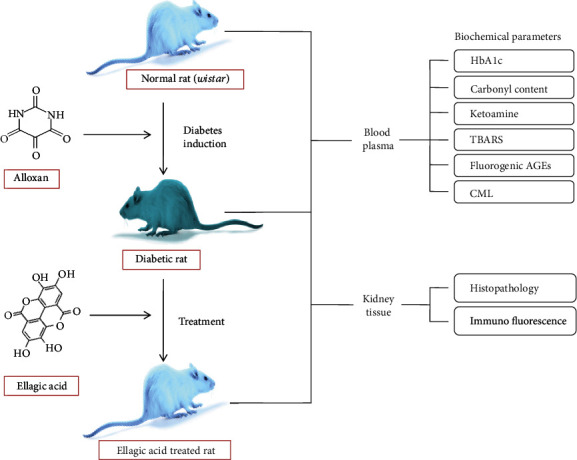
Overall protective effects of EA against alloxan-induced diabetes in Wistar rats.

The kidney tissues of control, diabetic, and EA-treated animals were analyzed for the detection of AGE-IgG immune complex deposition. To witness the anatomical changes in the kidney of all group animals, sections of kidney tissues were screened by light microscopy using hematoxylin and eosin stain (H&E staining) [22]. The hyperglycaemic condition promotes AGE-LDL-IgG-IC deposition in the renal organ [31]. Furthermore, the immunochemical evaluation was performed to detect the presence of AGE-LDL-IgG immune complex deposition in the kidney of normal, diabetic, and diabetic EA-treated group of male Wistar rats by using the fluorescence microscopy [38, 39]. All the animal-related experiments are represented in Scheme 1.

### 2.10. Data Analysis

Data are expressed as mean ± SD. Significant difference between treatments was defined at a level of *P* < 0.05 and *P* < 0.01. Statistical significance was determined using GraphPad Prism 5. Analysis of variance and Duncan's test were used to evaluate data. Values for ^∗^*P* < 0.05 were considered significant and those of ^∗∗^*P* < 0.01 were considered highly significant.

## 3. Results and Discussions

### 3.1. Ellagic Acid (EA) Toxicity Test and Administration Dose Levels

Owing to the implications of ellagic acid (EA) in distinct applications, the current *in vivo* study was premeditated not only to evaluate the antidiabetic effects of EA but also to assess its protective impact on AGE-induced pathologies in alloxan-induced diabetes rats. Assessment of the toxicity of therapeutic agents in various *in vitro* and *in vivo* systems is the prerequisite for the drug discovery program [40]. Therefore, before moving to the therapeutic interventional analysis, we assessed the toxic effects of EA when administered with varying doses (50, 100, and 150 mg kg^−1^ b.w.) in rats. Careful observation of the animals depicted that all these concentrations of EA were safe as no death, abnormal behaviour/activity, or irritations were observed in EA-administered rats, when compared to normal control rats. Our findings are well in agreement with another report which demonstrated that administration of EA at much higher doses did not show any mortality or treatment-related clinical signs [41]. Another study concluded that the treatment of diabetic rats with EA at 10 and 30 mg kg^−1^ b.w. is safe and did not lead to any significant changes in the activities of animals [42]. Conversely, EA has also showed protective effects against drug-induced toxicity [42]. Therefore, we selected 10 and 20 mg kg^−1^ b.w. of EA for the assessment of protective effects of EA against alloxan-induced diabetes in rats.

### 3.2. LDL Glycation Inhibition by Ellagic Acid

**Figure 1:**
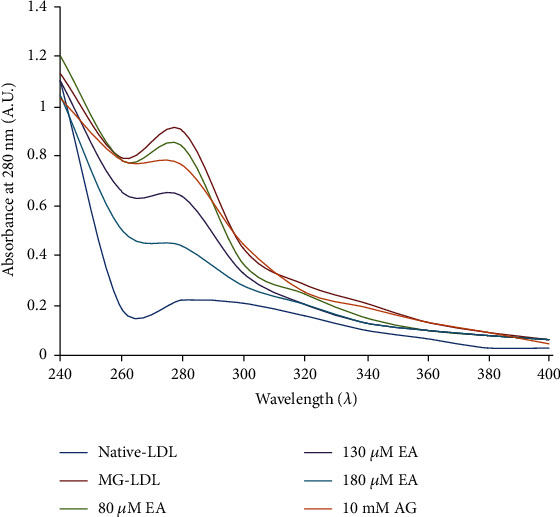
Effect of ellagic acid (EA) on LDL glycation induced change in the hyperchromicity pattern. The decrease in absorption was observed, as the concentration of EA increases from 80, 130, and 180 *μ*M in glycated LDL samples. Each value is mean ± SD for three experiments. The ^∗^*P* < 0.05 and ^∗∗^*P* < 0.01 values differ significantly with MG-LDL- and AG-treated samples.

Our results from UV-Vis spectroscopic analysis demonstrated that structural perturbation of MG-glycated LDL increases absorbance (0.9 ± 0.03 nm) which cause a 56% increase in hyperchromicity as compared to the absorbance of native LDL conformers (0.224 ± 0.02 nm) as shown in the absorption spectra at 280 nm (Figure 1). This enhanced absorbance significantly (*P* < 0.01) decreases after incubation of MG-LDL with EA at varying concentrations. EA in the glycation mixture showed % inhibition of glycation which was observed at 10% (0.84 ± 0.09 nm), 37% (0.6 ± 0.02 nm), and 56% (0.42 ± 0.04 nm) at the concentrations of 80, 130, and 180 *μ*M of EA-treated glycated LDL (Table 1). However, glycated samples treated with standard AG (10 mM) exhibit less inhibitory action against glycation as compared to EA (Figure 1). These ameliorative effects might have been accompanied by the interference of the aforesaid compounds with the initial attachment of MG to the amino groups of proteins, therefore blocking the formation of MG-LDL. There is growing evidence that production of ROS increased in diabetes patients, and the oxidative stress is associated with hyperglycaemia and diabetic complications [40, 43]. The ketoamine adduct formation, a marker of early glycation, could be inhibited by EA indicating that EA could exert their effect at the early stage of glycation. However, some EA derivatives are superior inhibitors to prevent glycation at the early, intermediate, and late stages to inhibit AGE formation [44–47]. Therefore, our in vitro LDL glycation and its treatment with EA results exhibited that susceptibility of LDL to glycation decreases significantly at concentrations of 180 *μ*M EA.

### 3.3. *In Vitro* and *In Vivo* Treatment of EA Decreases Ketoamines

**Figure 2:**
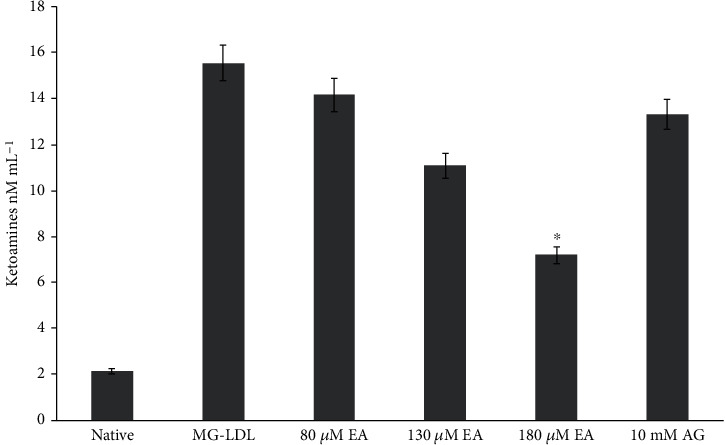
Effect of EA on MG-LDL induced ketoamines *in vitro*. Each value is mean ± SD for three determinations. The ^∗^*P* < 0.05 values differ significantly with MG-LDL- and AG treated samples.

**Figure 3:**
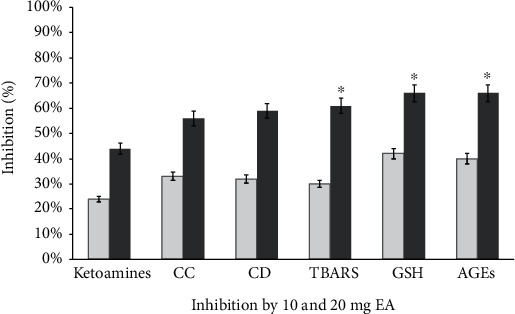
Effect of oral administration of 10 (□) and 20 (■) mg of EA (groups III and IV) causes % change in ketoamine, carbonyl content (CC), conjugated dienes (CD), TBARS, GSH, and AGE levels in alloxan induced diabetic Wistar rats *in vivo*. The ^∗^*P* < 0.05 values differ significantly with diabetic rat plasma components estimated in the figure for diabetic EA-treated rat model.

In vitro glycated LDL showed an 86% increase in the Schiff base product fructosamine (1-amino-1-deoxy fructose), which is a stable ketoamine (15.31 ± 1.31 nM mg^−1^) as compared with native LDL (2.1 ± 0.23 nM mg^−1^). The glycation reaction with 80 and 130 *μ*M EA showed the inhibitory role of glycation by the decreasing rate of fructosamine content (14.3 ± 0.4 nM mg^−1^ and 11.4 ± 0.3 nM mg^−1^) which is 11.36% and 34.41%, respectively (with no significant difference). However, ketoamine moiety decrease (7.2 ± 0.5 nM mg^−1^) in 180 *μ*M EA-treated antiglycation mixture is 52%, showing strong inhibition of early glycation products that have low ketoamine content (significant difference with 10 mM AG) (Figure 2 and Table 1). However, *in vivo* animal model group II diabetic rats showed 61% increase in ketoamine-LDL adducts (29.3 ± 1.6 nM mL^−1^) of plasma as compared with group I healthy control rat plasma ketoamine (8.34 ± 1.1 nM mL^−1^). The diabetic rats with 10 mg EA (group III) dose showed the inhibitory effect that showed a decrease in plasma ketoamine (22.1 ± 2.1 nM mL^−1^) by 24% with 20 mg EA (group IV) by 44% (with significant difference) decrease in plasma ketoamine content (16 ± 0.3 nM mL^−1^) (Figure 3). Ellagitannins (ETs), EA, and its metabolites exhibited similar great potential for the treatment of oxidative stress-mediated human diseases. AGE inhibitors prevented the formation of AGEs at the late stage of glycation, but a few of them exerted their effects at its early stage [48]. Therefore, the NBT assay results suggested that the decrease in early glycation product ketoamines by EA may be due to inhibition of LDL glycation with MG.

### 3.4. Protein-Carbonyl Content Trapping by EA

**Figure 4:**
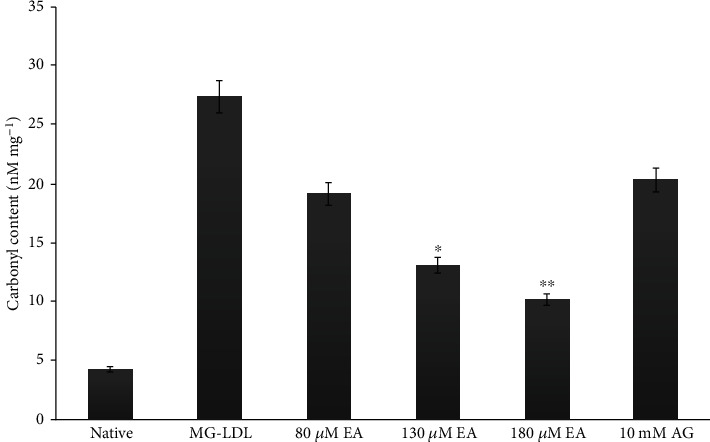
Effect of EA on MG-LDL induced carbonyl content *in vitro*. Each value is mean ± SD for three determinations. The ^∗^*P* < 0.05 values differ significantly with MG-LDL- and AG-treated samples.

The elevated protein-carbonyls in glycated LDL (27.3 ± 0.3 nM mg^−1^) sample were recorded as 78% high as compared to that in the native LDL sample (4.24 ± 0.5 nM mg^−1^). In our *in vitro* antiglycation experiments, little inhibitory effect of EA was observed at 80 *μ*M (19.1 ± 0.2 nM mg^−1^) which is 17.84% as compared to 10 mM AG (with no significant difference). However, 130 *μ*M EA (13.2 ± 0.4 nM mg^−1^) and 180 *μ*M EA (10 ± 1 nM mg^−1^) concentrations showed 43.43% and 62% inhibition, respectively, of carbonyl content as compared to AG (with significant difference) (Figure 4 and Table 1). Furthermore, at the carbonyl content estimation *in vivo*, group II diabetic animal plasma carbonyl increases, recorded as a 72% significant increase in carbonyl content (24 ± 2.1 nM mL^−1^) as compared to the carbonyl content of normal control group I animal plasma (5.4 ± 1.2 nM mL^−1^). The carbonyl content scavenging event in EA-treated group III (16 ± 0.80 nM mL^−1^) and group IV animal (10.4 ± 1.1 nM mL^−1^) plasma exhibited 33% and 56% low level of carbonyl content as compared to diabetic control group II, respectively. Several phenolic compounds and phenol polymers showed significant inhibitory effects on the formation of AGEs. Their antiglycation activities were not only brought about by their antioxidant activities but also related to their trapping abilities of RCS such as dicarbonyls, an intermediate reactive carbonyl of AGE formation [49, 50]. These results advocate that EA could inhibit LDL glycation by restricting the attack of dicarbonyls. EA could inhibit the *α*-dicarbonyl compounds in a concentration-dependent manner [51]. According to Muthenna et al., glycation-mediated damage to protein was also prevented by EA as there was a 50–90% reduction in the formation of protein-carbonyls upon glycation in the presence of a 100–150 *μ*M concentration [51].

### 3.5. Intrinsic Fluorescence Spectra *In Vitro*

**Figure 5:**
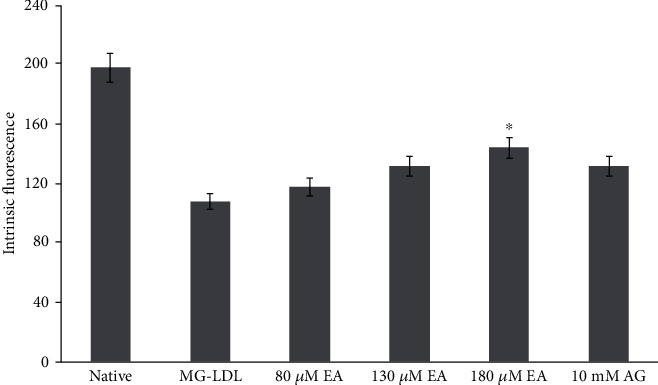
Effect of EA on intrinsic fluorescence of glycated LDL. Each value is mean ± SD for three experiments *in vitro*. The ^∗^*P* < 0.05 and values differ significantly with MG-LDL- and AG-treated samples.

To confirm the antiglycative role of EA, intrinsic fluorescence of native, glycated MG-LDL and EA and AG treated MG-LDL sample was measured. Glycation of LDL by MG results in intrinsic fluorescence quenching, due to glycation mediated structural changes and perturbation in protein aromatic amino acid microenvironment [22]. All the samples were excited at 295 nm, which is specific for tryptophan residues and showed strong fall of emission peak of glycated LDL (108 ± 16) as compared to native LDL peak (191 ± 16) at 346 nm (Figure 5). Therefore, AGE-LDL exhibit loss of fluorescence intensity (54%) compared to native LDL (Table 1). However, the protective role of 80, 130, and 180 *μ*M of EA with significant increase in fluorescence intensity (118 ± 7, 132 ± 9, and 144 ± 6) is 19%, 31%, and 40%, respectively. However, AG also showed increase in intrinsic fluorescence by 24% in glycated LDL sample (Figure 5). Tyrosine (Tyr) modification and lipid peroxidation appear to disturb hydrophobic stacking of aromatic amino acids in the LDL core protein and LDL phospholipids belt [52]. Therefore, EA was fortifying against these structural changes which was evident from the increase in intrinsic fluorescence intensity of glycated LDL treated with higher EA concentrations (130 and 180 *μ*M) which is significantly much greater than AG-treated glycated LDL.

### 3.6. Antiglycation-Inhibition of AGE Formation

**Figure 6:**
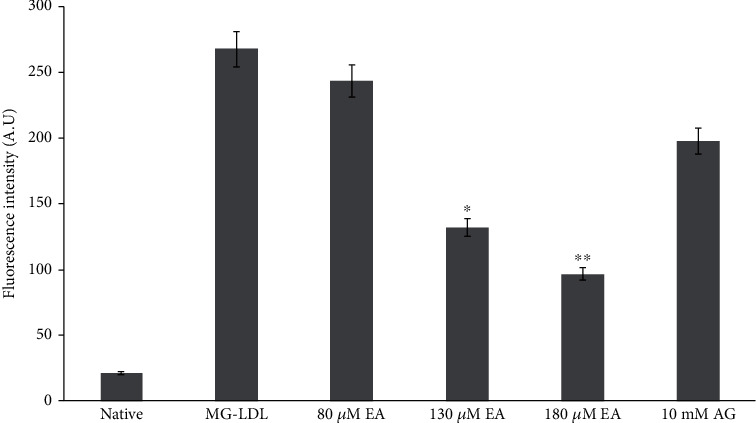
Effect of EA on glycated LDL induced AGEs *in vitro*. Each value is mean ± SD for three experiments. The ^∗^*P* < 0.05 and ^∗∗^*P* < 0.01 values differ significantly with MG-LDL- and AG-treated samples.

Fluorescence spectrometry was done to detect the presence of AGEs in glycation and antiglycation mixture. EA-treated glycated LDL mixture exhibits decrease in fluorescence intensity as compared to the glycated LDL (AGE-LDL) sample which is recorded to be 13.5, 54.4, and 70% (Table 1). However, a well-known AGE inhibitor AG (10 mM) showed nonsignificant decrease in AGE-LDL when compared with the glycated LDL sample (Figure 6). Increase in fluorescence intensity was observed in diabetic control group II rats when compared to its healthy control group I. The decrease in AGE-specific intensity is 40% and 62% in EA groups III and IV, respectively, which suggested that the antiglycation event of EA could inhibit the formation of AGEs (Figure 3 and Table 2). MG and GO are well-known precursors of AGEs, and trapping reactive carbonyl content may reduce the formation of AGEs [53]. Serum levels of AGEs increased and are correlated with type II diabetes mellitus and coronary heart disease [54].

Previous studies on bioactive compound role in antiglycation suggests these agents play a role in delaying the AGE formation through the prevention of oxidation of glucose and Amadori product [55]. The investigations in relation with AGEs inhibitors would offer a potential therapeutic approach for the prevention of diabetic or other pathogenic complications. Several inhibitors can suppress AGE formation by scavenging certain precursors such as 1,2-dicarbonyls. An evaluation of direct MG-trapping capacity suggested, seed extracts could directly scavenge carbonyl compounds [56, 57]. Both synthetic compounds and natural products have been evaluated as inhibitors against the formation of AGEs. So far, phenolic antioxidants such as EA, have been found to be the most promising agents, and their activities against AGE formation to correlate highly with their reactive carbonyl inhibition as well as those which possess AGEs inhibition capacities [51, 56].

### 3.7. EA-Treated Glycated LDL Eletrophoretic Mobility

**Figure 7:**
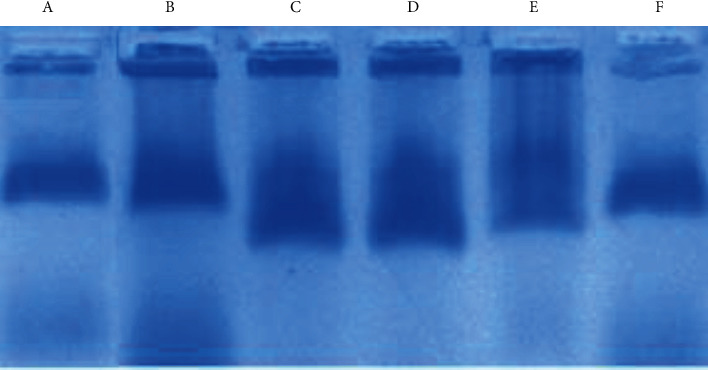
Agarose gel electrophoresis of plasma LDL (lane A), native LDL (lane B), and MG-LDL (lane C) while lanes D, E, and F show a banding pattern of MG-LDL with 80, 130, and 180 *μ*M EA-treated samples. The banding pattern shows significant electrophoretic mobility change from lanes E to F as compared to MG-LDL (lane C).

The LDL molecules were stained with Sudan black for the visualization of LDL molecule electrophoretic mobility in the gel. The agarose gel electrophoresis pattern of MG-glycated LDL suggested that that glycation can change the electrophoretic mobility and anodic migration of LDL when compared to the native LDL molecule (Figure 7) [22]. Glycated LDL migrated apparently faster than did plasma LDL and native LDL toward the positively charged anode. The faster migration might be due to glycation-induced damage in the structure of LDL molecules which make it shorter and lighter. However, glycated LDL treated with 80 *μ*M EA did not show any significant change in the migration of LDL as compared with glycated LDL. The decrease in electrophoretic mobility change was observed in 130 and 180 *μ*M EA-treated glycated LDL as compared with the glycated LDL sample, which points towards the chances of less structure modification and alteration of LDL molecule in the presence of EA (Figure 7).

### 3.8. FBS and HbA1c in EA-Treated Diabetic Animals

Alloxan dose injection (65 mg/kg b. wt.) to healthy Wistar rats was found to enhance extremely elevated hyperglycaemia followed by slight reduction in their body weight. Furthermore, the statistical significant increase in FBS (271 ± 23 mg dL^−1^) and HbA1c (6.77 ± 0.12%) of diabetic group II-IV rats as compared to the FBS (77 ± 9 mg dL^−1^) and HbA1c (4.7 ± 0.2%) of group I healthy control rats. However, the alloxan-induced diabetic groups III-IV treated with a dose of EA (10 and 20 mg/kg b.wt.) showed significant reduction in FBS of groups III and IV (181 ± 11 and 136 ± 09 mg dL^−1^) and HbA1c of groups III and IV (5.7 ± 0.1% and 5.2 ± 0.16%) after 25 days of treatment as compared to the diabetic rat group II (Table 3). Inhibition of catalytic activities of both *α*-glucosidase and *α*-amylase could delay and prolong digestion of overall carbohydrates, leading to the retardation of glucose adsorption and consequently blunting in blood sugar level [58–60]. EA prevents the interaction of the N-terminal amino acid valine of the *β*-chain of human hemoglobin with sugar carbonyls to form an initial Amadori product, thereby inhibiting the following formation of vigorous HbA1c [21, 34]. Our findings suggest and reveal the inhibition of nonenzymatic glycation as evidenced by decrease in the HbA1c level of EA-treated diabetic animals.

### 3.9. GSH, CD, and TBARS Estimation: The Preventive Role of Ellagic Acid

There is an observable percent change in reduced GSH level in plasma diabetic EA-treated rats. Therefore, diabetic group II rat plasma GSH level was21.43 ± 6.8 nm mL^−1^ which was a significant decrease compared to group I normal control rat plasma GSH level of 46.1 ± 5.1 nm mL^−1^. Furthermore, group III (10 mg EA) and IV (20 mg EA) rats with exposure to EA caused a significant increase in the reduced GSH level. Therefore, EA dosing in diabetic rats significantly increase by 32.23 ± 3.10 nM mL^−1^ and 37.11 ± 4.10 nM mL^−1^ the plasma GSH content (Table 2). The preventive role of EA results increase (42 and 66%) in the GSH content as compared to diabetic group II (Figure 3). GSH is involved in direct scavenging of free radicals by virtue of its thiol group.

To estimate lipid peroxidation level, trace amounts of CD were also detected from the lipid content of all four groups. CD content of group II rats was significantly higher (17.7 ± 1.2 nM mL^−1^) than that of control group I (10.42 ± 0.16 nM mL^−1^). However, the level of CD in groups II and IV was increased significantly. Compared with diabetic control group II rats (17.7 ± 1.2 nM mL^−1^), group III which was treated with EA, i.e., 10 mg EA-treated animal plasma CD level, was decreased (32%) moderately (15.1 ± 1.40 nM mL^−1^) while significant decrease (59%) in concentration of CD (13.36 ± 0.51 nM mL^−1^) was found in group IV with 20 mg EA-treated diabetic rat plasma (Figure 3 and Table 2). The generation of MG-induced hydroxyl radicals was measured as MDA by thiobarbituric acid-reactive substance (TBARS). Diabetic rat group II plasma level of TBARS (1.8 ± 0.06 nM mL^−1^) was much higher than group I normal control rats (0.3 ± 0.03 nM mL^−1^). The significant percent decrease of TBARS in plasma of EA-treated group III (10 mg) (1.22 ± 0.2 nM mL^−1^) and group IV (20 mg) (0.66 ± 0.4 nM mL^−1^) (Figure 3 and Table 2). The *Emblica officinalis* and its active constituent, EA, exert antidiabetic activity significantly increasing plasma total GSH and decreasing TBARS in diabetic rats [60]. Therefore, our results also suggested that EA might be involved in enhancing the activity or expression of glutathione peroxidase (GPx) which helps to increase the level of reduced GSH and decrease TBARS in EA-treated diabetic rats. Previously, it was suggested by one study that investigated the antioxidant effect of EA that it counteracted the effect in diabetic animal model and also exhibited a significant increase in the antioxidants such as the enzyme GPX level [21].

### 3.10. Reduction in AGE-CML

**Figure 8:**
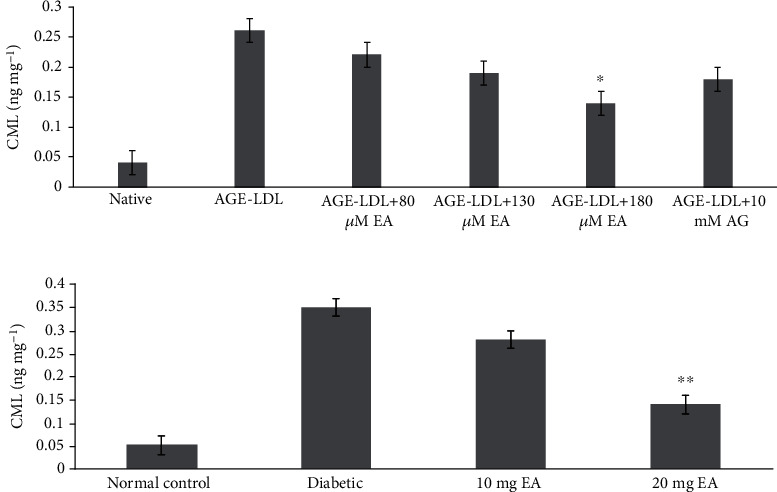
(a) CML detection in native, AGE-LDL- and AGE-LDL EA-treated samples *in vitro*. (b) Group I normal control, group II diabetic control and group II-IV was 10 and 20 mg EA-treated diabetic rats. Each value is mean ± SD for three experiments. The ^∗^*P* < 0.05 and ^∗∗^*P* < 0.01 values differ significantly with diabetic group II rats.

*In vitro* EA-treated MG-LDL level of CML was significantly decreased in 130 and 180 *μ*M EA-treated samples which were 0.211 ± 0.023 and 0.145 ± 0.035 ng mL^−1^, respectively, as compared to MG-LDL (0.249 ± 0.039 ng mL^−1^). Native LDL sample CML level was 0.039 ± 0.015 ng mL^−1^ (Figure 8(a)). The glycation of LDL can cause accumulation of carbonyl content (MG, GO, and 3-DG) which can further react to proteins, and the end results of chronic glycation can ultimately cause formation of heterogeneous AGEs and their precursors such as CML. However, in diabetic and EA-treated animal model, CML detection was done in separated animal sera from groups I–IV. The levels of CML significantly decreased in the EA-treated diabetic animals of group III and group IV were 0.283 ± 0.043 and 0.142 ± 0.031 ng mL^−1^ sera as compared to the diabetic animal group II (0.344 ± 0.043 ng mL^−1^). Group I CML level was 0.099 ± 0.025 ng mL^−1^ sera (Figure 8(b)). Glycation of protein with reactive carbonyls (MG, GO, and 3-DG) leads to formation of intermediate reactive carbonyls (MG, GO, and 3-DG) and AGEs. These reactive carbonyls can further glycate the proteins with the same intensity and induce carbonyl stress and AGEs. Our results of CML inhibition by EA are correlated with the study in which EA inhibited the accumulation of CML in the diabetic kidney [61]. The antiglycation studies of EA suggested that EA could protect the proteins to some extent against the formation of AGEs induced by glycation as evident by the progressive decrease in AGEs with increasing EA concentration [51, 62].

### 3.11. AGE-IgG Immune-Complex (IC) Detection

**Figure 9:**
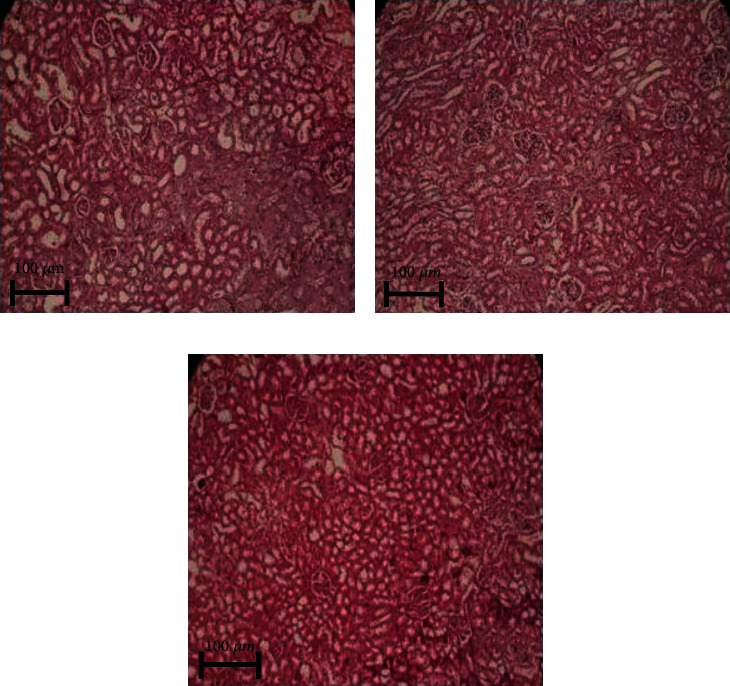
Histopathology study of diabetic and EA-treated rats. (a) Normal control rats showing kidney with normal histological structures and (b) diabetic control showed narrowing of glomeruli, and GBM thickening damaged histological structures, while EA-treated diabetic rats (c) observed vascular congestion, glycogen deposition, narrowing in the bowman space, and reduced thickening of GBM as compared to the diabetic group II rats.

**Figure 10:**
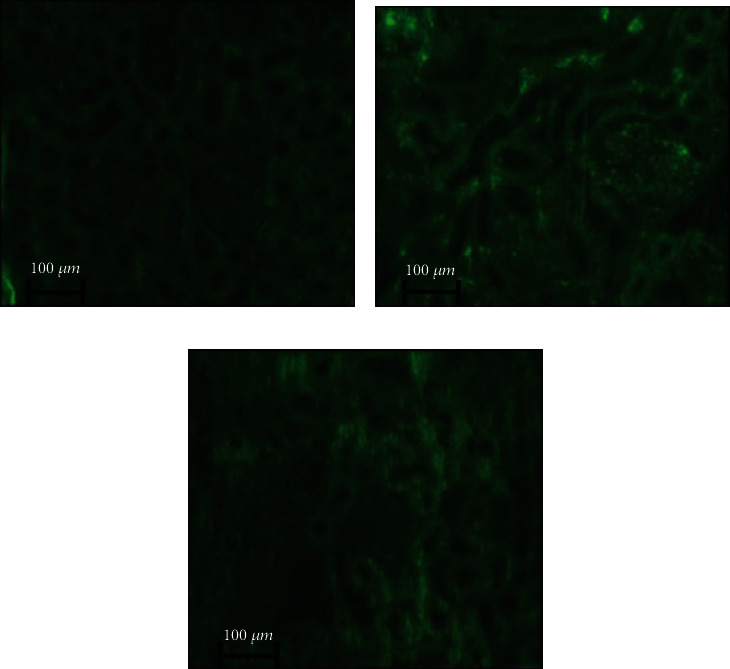
Immunochemical study reveals AGE-IgG deposition in diabetic and EA-treated animal kidney tissues. (a) Normal control rat showing no immunoreactivity. (b) Diabetic control rat kidney tissue shows intense staining and immunoreactivity (AGE-IgG) in the glomerulus. (c) EA-treated diabetic rat kidney showed less intense staining and immunoreactivity of glomerulus same as the normal control group I.

The glycation of LDL increases LDL-AGEs with antigenic epitopes to induce autoantibody production that will lead to the accumulation AGE-IgG immune complexes [10, 39]. The accelerated AGE-IgG-IC accumulation creates oxidative stress in the kidney of diabetic rat which may induce kidney lesion [22, 39, 63, 64]. The characteristic changes were observed in the glomerulus such as thickening of mesangial cells and glomerular basement membrane (GBM) of kidney tissue in diabetic group II rats (Figure 9(b)). Interestingly, 20 mg EA-treated group IV rat kidney revealed partial glomerular damage and less morphological changes (Figure 9(c)) as the control group I rat kidney section (Figure 9(a)). Furthermore, immunochemistry study results revealed a strong staining for immunoreactivity-mediated deposition of AGE-IgG immune complex (IC) in the renal tubules and the glomeruli of group II diabetic rat kidney section (Figure 10(b)). The immunochemistry of group IV with 20 mg EA-treated rat kidney tissue exhibits weak fluorescence intensity, which concluded less number of AGE-IgG-IC deposition in GBM. Our results suggested that EA can improves renal structure and function (Figure 10(c)) comparatively similar as the group I control (Figure 10(a)). However, the GBM thickening of kidney in experimental models imply not only deposited but also soluble immune complexes which can also damage glomeruli of the kidney [65, 66]. Therefore, the immunofluorescence experiment reveals a preventive role of EA against glomerular damage mediated by the structural irregularities in the kidneys under diabetic condition providing supportive evidence of the nephroprotective activity of EA. These observations from our study suggest a clinical use of bioactive natural compounds for the prevention of metabolic dysfunction by improving the free radical defense system and its antiglycative effect.

## 4. Conclusion

The dietary intervention and the use of functional foods have been shown to have an important role in the management of diabetes and its complications. However, many studies on functional foods are centered on antioxidant, hypoglycaemic, and anti-inflammatory effects but not focused on the antiglycating activity. The results of the present study are to provide a scope for controlling diabetic pathological conditions through food sources that are rich in EA.

In this study, we evaluate the low concentration of EA as an antiglycating compound to scavenge and inhibit glycating agents such as MG and glycation products in the glycation model *in vitro* as well as *in vivo* animal model. Therefore, the phenolic EA have been found to be the most promising agents for antiglycation process which correlate with their antioxidant potential which decreases the plasma GSH, MDA, and CD concentration in diabetic animals. Accumulated ROS is responsible for oxidation of polyunsaturated fatty acids that result in MDA formation. The formation of MDA is used as an indirect biomarker of oxidative stress in tissues. Our results suggested that ellagic acid may exert its protective effect against alloxan-induced diabetes by decreasing lipid peroxidation and by inhibition of early, intermediate products and AGE formation from glycation. The accumulation of glycated LDL-AGEs may cause autoimmune response and that may lead to accumulation of LDL-AGE-specific IgG. Furthermore, these AGE-IgG-immune complexes may be deposited in the renal tissues. The antiglycative potential of EA helps in inhibition of AGE-LDL immune complex deposition in GBM of rat kidney investigated by histopathological and immunostaining methods correlated with the decrease rate of progression of diabetes. In conclusion, fruits like berries and pomegranate and other vegetables might be a source of EA and a potential natural resource of novel dietary phytonutrients. The effect of pure supplement of EA provides scavenging and inhibitory activities against dicarbonyl compounds, MDA, and AGE inhibition activity, which could be helpful in inhibition of glycation and diabetes treatment. Based on these beneficial effects of EA in alloxan-induced diabetic rats, we concluded that EA can be used in preventive or complementary medicine for the treatment and management of diabetes and its associated complications.

## Figures and Tables

**Table 1:** Characterization of ellagic acid- (EA-) treated MG glycated LDL *in vitro*.

Characterization	Native LDL	MG-LDL	MG-LDL + 80 *μ*M EA	MG-LDL + 130 *μ*M EA	MG-LDL + 180 *μ*M EA	Maximum % inhibition by EA (180 *μ*M)
UV-absorbance (280 nm)	0.224 ± 0.02	0.9 ± 0.03	0.84 ± 0.09	0.6 ± 0.02^∗^	0.42 ± 0.04^∗∗^	56%
Fluorescent AGEs	21.30 ± 2	268 ± 11	243 ± 07	132 ± 9^∗^	96 ± 10^∗∗^	70%
Ketoamines nM mg^−1^	2.1 ± 0.23	15.3 ± 1.3	14.3 ± 0.4	11.4 ± 0.3	7.2 ± 0.5^∗^	52%
Carbonyl content (CC) nM mg^−1^	4.24 ± 0.5	27.3 ± 0.3	19.1 ± 0.2	13.2 ± 0.4^∗^	10 ± 1^∗^	62%
Intrinsic fluorescence (A.U.)	191 ± 16	108 ± 16	118 ± 7	132 ± 9	144 ± 6^∗^	40%

Data shown in table represents average of three experiments. The values represent the mean ± SD. The ^∗^*P* < 0.05 and ^∗∗^*P* < 0.01 values differ significantly with MG-LDL.

**Table 2:** Glycation inhibition from EA treatment characterized by biochemical estimations in alloxan-induced diabetic rats *in vivo*.

Groups	Ketoamines (nM mL^−1^)	CC (nM mL^−1^)	TBARS (nM mL^−1^)	CD (nM mL^−1^)	AGEs (A.U.)
Group I control	8.34 ± 1.1	5.45 ± 1.2	0.3 ± 0.03	10 ± 0.16	10 ± 2
Group II diabetic control	29.3 ± 1.6	24 ± 2.1	1.8 ± 0.06	17.7 ± 1.2	116 ± 10
Group III diabetic + 10 mg EA	22.1 ± 2.1	16 ± 0.8	1.22 ± 0.2	15 ± 1.40^∗^	69 ± 8^∗^
Group IV diabetic + 20 mg EA	16 ± 0.3^∗^	10.4 ± 1.1^∗∗^	0.66 ± 0.03^∗^	13.36 ± 0.2^∗^	43 ± 5^∗∗^
% inhibition by 10 mg EA	24%	33%	32%	30%	40%
% inhibition by 20 mg EA	44%	56%	59%	61%	62%

Each value is mean ± SD. Five rats per cage in each group. The ^∗^*P* < 0.05 and ^∗∗^*P* < 0.01 values differ significantly with the diabetic group II.

**Table 3:** Effect of EA on FBS and HbA1c in diabetic rat model *in vivo*.

Groups	FBS (mg dL^−1^)	HbA1c (%)
Group I control	77 ± 9	4.7 ± 0.2
Group II diabetic control	271 ± 23	6.77 ± 0.12
Group III diabetic + 10 mg EA	181 ± 11	5.7 ± 0.1
Group IV diabetic + 20 mg EA	136 ± 09^∗^	5.2 ± 0.16^∗^
% inhibition by 10 mg EA	28%	21%
% inhibition by 20 mg EA	37%	47%

Each value is the mean ± SD for five rats in each group. The ^∗^*P* < 0.05 values differ significantly with the diabetic group.

## Data Availability

Data is available within the manuscript.

## Abbreviations

AGEs: Advanced glycation end products
EA: Ellagic acid
FBS: Fasting blood sugar
GSH: Glutathione
HbA1c: Hemoglobin A1c
LDL: Low-density lipoprotein
MG: Methylglyoxal
MDA: Malondialdehyde
AG: Aminoguanidine.

## References

[1] Zheng Y., Ley S. H., Hu F. B. (2018). Global aetiology and epidemiology of type 2 diabetes mellitus and its complications. *Nature Reviews Endocrinology*.

[2] Ahmad S., Khan H., Siddiqui Z. (2018). AGEs, RAGEs and s-RAGE; friend or foe for cancer. *Seminars in Cancer Biology*.

[3] Ahmad S., Khan M. Y., Rafi Z. (2018). Oxidation, glycation and glycoxidation--the vicious cycle and lung cancer. *Seminars in Cancer Biology*.

[4] Younus H., Anwar S. (2016). Prevention of non-enzymatic glycosylation (glycation): implication in the treatment of diabetic complication. *International Journal of Health Sciences*.

[5] Nishizawa Y., Koyama H., Inaba M. (2012). AGEs and cardiovascular diseases in patients with end-stage renal diseases. *Journal of Renal Nutrition*.

[6] Ahmed U., Thornalley P. J., Rabbani N. (2014). Possible role of methylglyoxal and glyoxalase in arthritis. *Biochemical Society Transactions*.

[7] Kizer J. R., Benkeser D., Arnold A. M. (2014). Advanced glycation/glycoxidation endproduct carboxymethyl-lysine and incidence of coronary heart disease and stroke in older adults. *Atherosclerosis*.

[8] Schleicher E., Deufel T., Wieland O. H. (1981). Non-enzymatic glycosylation of human serum lipoproteins elevated *ϵ*-lysine glycosylated low density lipoprotein in diabetic patients. *FEBS Letters*.

[9] Lyons T. J. (1993). Glycation and oxidation: a role in the pathogenesis of atherosclerosis. *The American Journal of Cardiology*.

[10] Khan M. Y., Alouffi S., Khan M. S., Husain F. M., Akhter F., Ahmad S. (2020). The neoepitopes on methylglyoxal (MG) glycated LDL create autoimmune response; autoimmunity detection in T2DM patients with varying disease duration. *Cellular Immunology*.

[11] Saad A. F., Virella G., Chassereau C., Boackle R. J., Lopes-Virella M. F. (2006). OxLDL immune complexes activate complement and induce cytokine production by MonoMac 6 cells and human macrophages. *Journal of Lipid Research*.

[12] Tames F. J., Mackness M. I., Arrol S., Laing I., Durrington P. N. (1992). Non-enzymatic glycation of apolipoprotein B in the sera of diabetic and non-diabetic subjects. *Atherosclerosis*.

[13] Corbett J. A., Tilton R. G., Chang K. (1992). Aminoguanidine, a novel inhibitor of nitric oxide formation, prevents diabetic vascular dysfunction. *Diabetes*.

[14] Wang Y., Qiu Z., Zhou B. (2015). *In vitro* antiproliferative and antioxidant effects of urolithin A, the colonic metabolite of ellagic acid, on hepatocellular carcinomas HepG2 cells. *Toxicology In Vitro*.

[15] Kerkeni M., Weiss I. S., Jaisson S. (2014). Increased serum concentrations of pentosidine are related to presence and severity of coronary artery disease. *Thrombosis Research*.

[16] Asif M. (2015). Antiglycation activity of vegetables aqueous and methanolic extracts. *Current Science Perspectives*.

[17] Degenhardt T. P., Alderson N. L., Arrington D. D. (2002). Pyridoxamine inhibits early renal disease and dyslipidemia in the streptozotocin-diabetic rat. *Kidney International*.

[18] Voziyan P. A., Hudson B. G. (2005). Pyridoxamine as a multifunctional pharmaceutical: targeting pathogenic glycation and oxidative damage. *Cellular and Molecular Life Sciences*.

[19] Brings S., Fleming T., Freichel M., Muckenthaler M. U., Herzig S., Nawroth P. P. (2017). Dicarbonyls and advanced glycation end-products in the development of diabetic complications and targets for intervention. *International Journal of Molecular Sciences*.

[20] Jurenka J. (2008). Therapeutic applications of pomegranate (Punica granatum L.): a review. *Alternative Medicine Review*.

[21] Altindağ F., Özdek U. (2021). Synergistic effects of sinapic acid and ellagic acid ameliorate streptozotocin-induced diabetic nephropathy by inhibiting apoptosis, DNA damage, and structural deterioration in rats. *Human & Experimental Toxicology*.

[22] Khan M. Y., Alouffi S., Ahmad S. (2018). Immunochemical studies on native and glycated LDL - an approach to uncover the structural perturbations. *International Journal of Biological Macromolecules*.

[23] Ahmad S., Akhter F., Moinuddin U., Shahab M. S. K. (2013). Studies on glycation of human low density lipoprotein: a functional insight into physico-chemical analysis. *International Journal of Biological Macromolecules*.

[24] Nabi R., Alvi S. S., Shah M. S. (2020). A biochemical & biophysical study on in-vitro anti-glycating potential of iridin against D-ribose modified BSA. *Archives of Biochemistry and Biophysics*.

[25] Alvi S. S., Nabi R., Khan M., Akhter F., Ahmad S., Khan M. S. (2021). Glycyrrhizic acid scavenges reactive carbonyl species and attenuates glycation-induced multiple protein modification: an in vitro and in silico study. *Oxidative Medicine and Cellular Longevity*.

[26] Ghisaidoobe A., Chung S. (2014). Intrinsic tryptophan fluorescence in the detection and analysis of proteins: a focus on Förster resonance energy transfer techniques. *International Journal of Molecular Sciences*.

[27] Ma'arfi F., Chandra S., Fatima J., Khan M. Y., Mir S. S., Yusuf M. A. (2020). Probing the structure–function relationship and amyloidogenic propensities in natural variants of apolipoprotein A-I. *Biochemistry and Biophysics Reports*.

[28] Noble R. P. (1968). Electrophoretic separation of plasma lipoproteins in agarose gel. *Journal of Lipid Research*.

[29] Macdonald Ighodaro O., Adeosun A. M., Adeboye Akinloye O. (2017). Alloxan-induced diabetes, a common model for evaluating the glycemic-control potential of therapeutic compounds and plants extracts in experimental studies. *Medicina*.

[30] Goswami S. K., Vishwanath M., Gangadarappa S. K., Razdan R., Inamdar M. N. (2014). Efficacy of ellagic acid and sildenafil in diabetes-induced sexual dysfunction. *Pharmacognosy Magazine*.

[31] Lin M. C., Yin M. C. (2013). Preventive effects of ellagic acid against doxorubicin-induced cardio-toxicity in mice. *Cardiovascular Toxicology*.

[32] King A. J. F. (2012). The use of animal models in diabetes research. *British Journal of Pharmacology*.

[33] Celik G., Semiz A., Karakurt S., Arslan S., Adali O., Sen A. (2013). A comparative study for the evaluation of two doses of ellagic acid on hepatic drug metabolizing and antioxidant enzymes in the rat. *BioMed Research International*.

[34] Akhter F., Alvi S. S., Ahmad P., Iqbal D., Khan M. S. (2019). Therapeutic efficacy of Boerhaavia diffusa (Linn.) root methanolic extract in attenuating streptozotocin-induced diabetes, diabetes-linked hyperlipidemia and oxidative-stress in rats. *Biomedical Research and Therapy*.

[35] Olar L. E., Ştefan R., Berce C., Ciobanu D., Papuc I. (2015). The fluorescence identification of advanced glycation end products in streptozotocin–induced diabetic rats’ plasma samples. *Bulletin UASVM Veterinary Medicine*.

[36] Sedlak J., Lindsay R. H. (1968). Estimation of total, protein-bound, and nonprotein sulfhydryl groups in tissue with Ellman's reagent. *Analytical Biochemistry*.

[37] Alvi S. S., Ansari I. A., Khan I., Iqbal J., Khan M. S. (2017). Potential role of lycopene in targeting proprotein convertase subtilisin/kexin type-9 to combat hypercholesterolemia. *Free Radical Biology & Medicine*.

[38] Ashraf J. M., Ahmad S., Choi I. (2015). Recent advances in detection of AGEs: immunochemical, bioanalytical and biochemical approaches. *IUBMB Life*.

[39] Akhter F., Khan M. S., Singh S., Ahmad S. (2014). An immunohistochemical analysis to validate the rationale behind the enhanced immunogenicity of D-ribosylated low density lipo-protein. *PLoS One*.

[40] Nabi R., Alvi S. S., Shah A. (2019). Modulatory role of HMG-CoA reductase inhibitors and ezetimibe on LDL-AGEs- induced ROS generation and RAGE-associated signalling in HEK-293 Cells. *Life Sciences*.

[41] Tasaki M., Umemura T., Maeda M. (2008). Safety assessment of ellagic acid, a food additive, in a subchronic toxicity study using F344 rats. *Food and Chemical Toxicology*.

[42] Anwar S., Younus H. (2017). Antiglycating potential of ellagic acid against glucose and methylglyoxal-induced glycation of superoxide dismutase. *Journal of Proteins & Proteomics*.

[43] Fakhruddin S., Alanazi W., Jackson K. E. (2017). Diabetes-induced reactive oxygen species: mechanism of their generation and role in renal injury. *Journal of Diabetes Research*.

[44] Urios P., Grigorova-Borsos A. M., Sternberg M. (2007). Flavonoids inhibit the formation of the cross-linking AGE pentosidine in collagen incubated with glucose, according to their structure. *European Journal of Nutrition*.

[45] Price D. L., Rhett P. M., Thorpe S. R., Baynes J. W. (2001). Chelating activity of advanced glycation end-product inhibitors. *Journal of Biological Chemistry*.

[46] Yin P., Yang L., Xue Q. (2018). Identification and inhibitory activities of ellagic acid- and kaempferol- derivatives from Mongolian oak cups against *α*-glucosidase, *α*-amylase and protein glycation linked to type II diabetes and its complications and their influence on HepG2 cells' viability. *Arabian Journal of Chemistry*.

[47] Rahbar S., Figarola J. L. (2003). Novel inhibitors of advanced glycation endproducts. *Archives of Biochemistry and Biophysics*.

[48] Mesías M., Navarro M., Gökmen V., Morales F. J. (2013). Antiglycative effect of fruit and vegetable seed extracts: inhibition of AGE formation and carbonyl-trapping abilities. *Journal of the Science of Food and Agriculture*.

[49] Wang W., Yagiz Y., Buran T. J., do Nascimento Nunes C., Gu L. (2011). Phytochemicals from berries and grapes inhibited the formation of advanced glycation end‐products by scavenging reactive carbonyls. *Food Research International*.

[50] Peng X., Cheng K., Ma J. (2008). Cinnamon bark proanthocyanidins as reactive carbonyl scavengers to prevent the formation of advanced glycation endproducts. *Journal of Agricultural and Food Chemistry*.

[51] Muthenna P., Akileshwari C., Reddy G. B. (2012). Ellagic acid, a new antiglycating agent: its inhibition of N*ϵ*-(carboxymethyl)lysine. *Biochemical Journal*.

[52] Khanam A., Alouffi S., Rehman S., Ansari I. A., Shahab U., Ahmad S. (2021). An *in vitro* approach to unveil the structural alterations in d-ribose induced glycated fibrinogen. *Journal of Biomolecular Structure and Dynamics*.

[53] Yoon S. R., Shim S. M. (2015). Inhibitory effect of polyphenols in Houttuynia cordata on advanced glycation end-products (AGEs) by trapping methylglyoxal. *LWT-Food Science and Technology*.

[54] Kiuchi K., Nejima J., Takano T., Ohta M., Hashimoto H. (2001). Increased serum concentrations of advanced glycation end products: a marker of coronary artery disease activity in type 2 diabetic patients. *Heart*.

[55] Povichit N., Phrutivorapongkul A., Suttajit M., Chaiyasut C., Leelapornpisid P. (2010). Phenolic content and in vitro inhibitory effects on oxidation and protein glycation of some Thai medicinal plants. *Pakistan Journal of Pharmaceutical Sciences*.

[56] Ahmad S. M., Ahmed N. (2006). Antiglycation properties of aged garlic extract: possible role in prevention of diabetic complications. *The Journal of Nutrition*.

[57] Reddy P. V., Beyaz A. (2006). Inhibitors of the Maillard reaction and AGE breakers as therapeutics for multiple diseases. *Drug Discovery Today*.

[58] Bhandari M. R., Jong-Anurakkun N., Hong G., Kawabata J. (2008). *α*-Glucosidase and *α*-amylase inhibitory activities of Nepalese medicinal herb Pakhanbhed (Bergenia ciliata, Haw.). *Food Chemistry*.

[59] Zhang J., Zhao S., Yin P. (2014). *α*-Glucosidase inhibitory activity of polyphenols from the burs of Castanea mollissima Blume. *Molecules*.

[60] Fatima N., Hafizur R. M., Hameed A., Ahmed S., Nisar M., Kabir N. (2017). Ellagic acid in *Emblica officinalis* exerts anti-diabetic activity through the action on *β*-cells of pancreas. *European Journal of Nutrition*.

[61] Raghu G., Jakhotia S., Reddy P. Y., Kumar P. A., Reddy G. B. (2016). Ellagic acid inhibits non-enzymatic glycation and prevents proteinuria in diabetic rats. *Food & Function*.

[62] Amor A. J., Gómez-Guerrero C., Ortega E., Sala-Vila A., Lázaro I. (2020). Ellagic acid as a tool to limit the diabetes burden: updated evidence. *Antioxidants*.

[63] Mitsuhashi T., Nakayama H., Itoh T. (1993). Immunochemical detection of advanced glycation end products in renal cortex from STZ-induced diabetic rat. *Diabetes*.

[64] Zhou B., Li Q., Wang J., Chen P., Jiang S. (2019). Ellagic acid attenuates streptozocin induced diabetic nephropathy via the regulation of oxidative stress and inflammatory signaling. *Food and Chemical Toxicology*.

[65] Barratt J., Eitner F. (2009). Sugars and immune complex formation in IgA nephropathy. *Nature Reviews Nephrology*.

[66] Wang X., Desai K., Clausen J. T., Wu L. (2004). Increased methylglyoxal and advanced glycation end products in kidney from spontaneously hypertensive rats. *Kidney International*.

